# Biofilm Growth on Different Materials Used in Contemporary Femoral Head Prosthesis: An In Vitro Study

**DOI:** 10.3390/jcm14051722

**Published:** 2025-03-04

**Authors:** Yonggyun Moon, Jaeyoung Hong, Sookyung Choi, Hyoungtae Kim, Hong Moon Sohn, Suenghwan Jo

**Affiliations:** 1 College of Medicine, Chosun University, Gwangju 61452, Republic of Korea; 2 Department of Orthopedic Surgery, Chosun University Hospital, Gwangju 61453, Republic of Korea

**Keywords:** periprosthetic joint infection (PJI), bacterial biofilm, femoral head prosthesis, *Staphylococcus aureus*, *Pseudomonas aeruginosa*

## Abstract

**Background/Objectives:** Periprosthetic joint infection (PJI) primarily results from bacterial biofilms adhering to prosthetic surfaces, making treatment challenging without prosthesis removal. This in vitro study aims to investigate whether the materials used in contemporary femoral head prosthesis influences bacterial biofilm development. **Methods**: Femoral head prostheses made of three different materials—cobalt–chrome, oxinium, and ceramic—were inoculated with either *Staphylococcus aureus* or *Pseudomonas aeruginosa* in separate experiments, with each pathogen tested independently. The samples were cultured under shaking conditions at 37 °C for 96 h to promote biofilm formation. Scanning electron microscopy (SEM) was used to confirm the presence of biofilms, and adherent biofilms were quantified by counting colony-forming units (CFUs) after sonication. Additionally, crystal violet staining was performed to assess biofilm distribution on the femoral head surfaces. Statistical analyses compared CFU counts across the different materials. **Results**: The mean CFU counts for *S. aureus* were 7.6 × 10^5^ ± 9.7 × 10^4^ for cobalt–chrome, 6.9 × 10^5^ ± 3.6 × 10^5^ for oxinium, and 1.1 × 10^6^ ± 3.0 × 10^5^ for ceramic femoral head prostheses. For *P. aeruginosa*, the CFU counts were 2.3 × 10^6^ ± 7.2 × 10^5^, 3.7 × 10^6^ ± 2.5 × 10^6^, and 2.2 × 10^6^ ± 8.9 × 10^5^, respectively. Regardless of the bacterial strain, differences among the three materials were within one log range, and no statistical significance was observed. While biofilms were confirmed using SEM, limited adherence was observed on the bearing surface, with the biofilm predominantly localized in the taper hole. **Conclusions**: The findings suggest that the material used in contemporary femoral head prostheses has minimal impact on bacterial biofilm formation. Surgeons’ choice of femoral head prosthesis material should base their material selection on factors other than PJI prevention.

## 1. Introduction

Periprosthetic joint infection (PJI) stands as one of the most devastating complications following arthroplasty, posing significant challenges to patient recovery and healthcare systems [1,2]. The importance of addressing PJI is underscored by recent epidemiological data from the American Joint Replacement Registry (AJRR), which identifies infection as the leading cause of revision surgery, accounting for 17.6% (or 20,742 cases) of all hip revision procedures [3]. With data collected on 1,317,887 hip arthroplasty procedures—including 1,014,772 total hip arthroplasties (THA) and 129,127 hip revision surgeries—this translates to an estimated 2% prevalence of PJI among patients who underwent THA. Despite advances in surgical techniques and postoperative care, the 10-year mortality rate for patients with PJI is reported to be 11.4%, which is significantly higher than the 2.2% mortality rate observed in patients without PJI, representing more than a fivefold increase in risk [4].

Difficulty in treating PJI is largely due to the formation of bacterial biofilms on the prosthetic surface [5]. Biofilms are complex communities of bacteria that adhere to surfaces and are encased in a protective extracellular matrix [6]. This matrix shields them from the host immune system and significantly increases antibiotic resistance, making systemic antibiotic treatment less effective [7]. As a result, antibiotic treatment alone is often insufficient, and the removal of the prosthesis is often necessary to eradicate biofilm-associated infections, which adds significant morbidity and increases healthcare costs [8].

Despite the existence of some studies that investigated influence of orthopedic materials on developing biofilm, the current body of research remains insufficient. Notably, previous studies have primarily used simplified models, such as machined discs, rather than actual femoral head prostheses, limiting their clinical relevance [9,10]. While these models share basic surface characteristics with implants, they fail to account for the complex geometry and structural features of actual prostheses, which can influence bacterial adhesion and biofilm formation.

Furthermore, although some investigations have employed actual implants, these studies typically focused on evaluating the antimicrobial resistance of biofilms retrieved from infected devices in patients with PJI, rather than cultivating biofilms on implants under controlled experimental conditions [11]. To address these gaps, the present study examines whether the material of the femoral head prosthesis influences biofilm development by assessing the biofilm-forming potential of *Staphylococcus aureus* and *Pseudomonas aeruginosa*—representative Gram-positive and Gram-negative bacteria frequently associated with PJI [12].

This study evaluates biofilm formation on three different materials used in contemporary femoral head prostheses—cobalt–chrome, oxinium, and ceramic—to determine whether any of these biomaterials exhibit superior resistance to bacterial colonization. Clinicians typically select femoral head materials based on factors such as mechanical properties, wear resistance, biocompatibility, and cost, aiming to optimize implant longevity and patient outcomes. Identifying material-dependent differences in biofilm susceptibility could provide valuable insights into the role of prosthesis material selection in PJI prevention. This knowledge may aid surgical decision making and contribute to improved patient outcomes.

## 2. Materials and Methods

### 2.1. Femoral Head Prostheses

**Figure 1:**
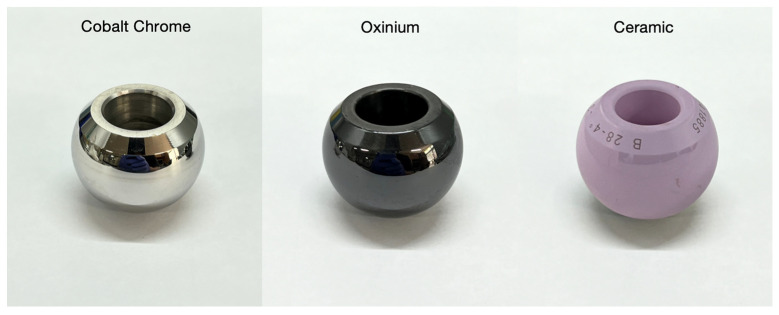
Images of femoral head implants utilized in the current study: cobalt–chrome, oxinium, and ceramic, respectively.

Femoral head made of either cobalt–chrome (Zimmer-Biomet, Warsaw, IN, USA), oxinium (Smith and Nephew, Watford, UK), and ceramic (Zimmer-Biomet, Warsaw, IN, USA) (Figure 1) were purchased from the local distributors and were utilized in the current study. Surface areas of each tested material were identical, and the diameter of these heads were 28 mm. All prostheses were developed to adopt a 14 mm diameter taper.

### 2.2. Bacterial Strains

*Staphylococcus aureus* (ATCC 29213) and *Pseudomonas aeruginosa* (ATCC 27853) were selected for the purpose of the current study, as they represent common Gram-positive and Gram-negative bacteria present in PJI [12].

### 2.3. Study Design

The bacterial culture process was standardized for both strains to ensure consistent biofilm formation. Initially, bacterial stocks were stored at −80 °C in glycerol-containing cryovials. For inoculation, 20 µL of the frozen bacterial stock was extracted and introduced into 20 mL of sterile culture medium (*S. aureus*: Tryptic Soy Broth [TSB] (MBcell, Seoul, South Korea), *P. aeruginosa*: Luria Bertani [LB] (MBcell, Seoul, Republic of Korea) broth). The cultures were incubated at 37 °C for 24 h under constant shaking at 200 rpm to allow bacterial growth to saturation [13,14].

After 24 h, 360 µL of the saturated culture was transferred into 360 mL of fresh sterile medium, which had been prepared according to the manufacturer’s recommended powder concentration, dissolved in distilled water, sterilized via autoclaving at 121 °C, 15 psi, for 15 min, and stored at 4 °C until use.

For *S. aureus*, the culture was incubated at 37 °C and 200 rpm for 6 h to reach the exponential growth phase [15]. Similarly, *P. aeruginosa* was incubated under the same conditions for 10 h to reach its respective exponential growth phase [16].

### 2.4. Biofilm Formation on Femoral Heads

Femoral heads made of cobalt–chrome (Zimmer-Biomet, Warsaw, IN, USA), oxinium (Smith and Nephew, Watford, UK), and ceramic (Zimmer-Biomet, Warsaw, IN, USA) (Figure 1) were utilized for biofilm formation. Each femoral head had an identical surface area and a uniform diameter of 28 mm, with a 14 mm diameter taper.

Prior to experimentation, all femoral heads were sterilized using an autoclave at 121 °C, 15 psi, for 15 min. Each femoral head was then placed in a sterile plastic container. These plastic containers were single-use, pre-sterilized biopsy containers, commonly utilized in surgical environments for storing tissue specimens. To ensure aseptic conditions, the containers were kept in their sterile packaging and opened only at the time of use.

Each plastic container was filled with 20 mL of the prepared bacterial suspension, and the samples were incubated at 37 °C for 24 h under constant shaking at 200 rpm to facilitate biofilm development [13,14]. During this period, the culture medium was replaced every 48 h, but the femoral heads and containers remained unchanged to preserve biofilm integrity. All experiments were performed in triplicate to ensure reproducibility.

Following the 96 h culture period, the femoral heads were subjected to the same autoclave sterilization process to ensure proper decontamination before disposal or further analysis.

### 2.5. Adherent Bacterial Quantification

The quantification process started with removing the planktonic bacteria, which was performed by removing the existing broth from the plastic container, adding normal saline, and gently agitating it 15 times to dislodge non-adherent bacteria. The femoral head was then transferred into 20 mL of fresh normal saline in a new plastic container and underwent sonication at 40 kHz for 10 min on ice to detach the biofilm from the femoral head surface [17,18]. Sonication was performed on ice to prevent heat generation from ultrasonic energy transfer, which could otherwise increase the sample temperature, potentially causing cell damage or altering sample properties. After sonication, the solutions were serially diluted and 50 µL aliquots were spread onto TSB or LB agar plates. These plates were then incubated at 37 °C for 24 h. Colony-forming units (CFUs) were counted for quantification of the detached biofilm.

**Figure 2:**
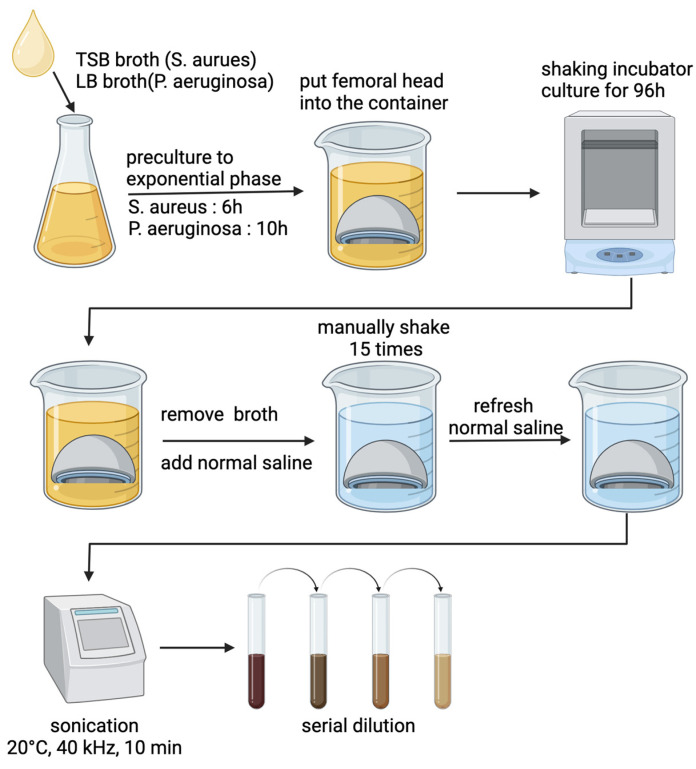
Summary of bacterial biofilm growth and quantification. Created in BioRender. Jo, S. (2025) https://BioRender.com/s05t629, accessed on 18 February 2025.

The protocol for developing and quantifying bacterial biofilm is summarized in Figure 2.

### 2.6. Scanning Electron Microscopy (SEM) Evaluation

Field-emission scanning electron microscope (FE-SEM, model SU8600) was used to confirm the formation of bacterial biofilm on femoral heads. For this process, femoral heads were sectioned to fit the SEM stage (height < 10 mm), during which the bearing surface of the femoral head was exposed for imaging. The biofilm on each material surface was grown by using the protocol described previously.

After allowing biofilm formation on the femoral heads, the samples were gently rinsed with phosphate-buffered saline (PBS) to remove non-adherent cells and were fixed in 2.5% glutaraldehyde at 4 °C for 24 h to preserve cellular structures. Following fixation, the samples were washed three times with PBS and dehydrated through a graded ethanol series (30%, 50%, 70%, 90%, and 100%) for 10 min each. Critical point drying was used to prevent the collapse of the biofilm structure due to surface tension [19]. Samples were oriented to ensure the smooth bearing surface faced upward during fixation process and SEM imaging.

### 2.7. Crystal Violet Staining

Crystal violet staining was used to determine the bacterial biofilm distribution on the femoral head surface. On the separate femoral heads, *S. aureus* biofilm was cultured as described previously. The samples were then immersed in 20 mL of 0.5% crystal violet solution and incubated at room temperature for 20 min for staining. The samples were gently washed in normal saline and air-dried at room temperature for 16 h [20].

In order to visualize the crystal violet staining efficiently, only femoral heads made of cobalt–chrome and ceramic materials were used, as oxinium heads, with their inherent dark color, posed challenges in accurately assessing the stain. However, even in the selected materials, the material properties of the femoral head prostheses made it difficult to clearly distinguish the stain in the original images. To address this limitation, images were captured in a controlled environment using a digital camera, and a false-color imaging method was employed to enhance contrast. RGB values of stained areas were manually sampled using a color-picking tool, and these values defined the color range for mask creation. A tolerance parameter was applied to account for variations in staining and lighting, and the cv2.inRange function in OpenCV generated a binary mask of the stained regions. A grayscale version of the image served as a neutral background, and the binary mask was overlaid with a fluorescent green (RGB: 0, 255, 0) color using cv2.addWeighted to combine the layers. This technique enhanced the visibility of biofilms while preserving structural details [21].

### 2.8. Statistical Analysis

Experiments were performed in triplicates, independently for materials and for bacterial strains, and repeated. The CFU counts for biofilm adherent among three materials for each bacterial species were reported as means with stand errors. The comparison was made using one-way analysis of variance (ANOVA) with a Bonferroni post hoc test (OriginPro, version 9.0). A *p*-value less than 0.05 was considered statistically significant, while mean value differences exceeding 3 logs were considered clinically significant [22].

## 3. Results

**Figure 3:**
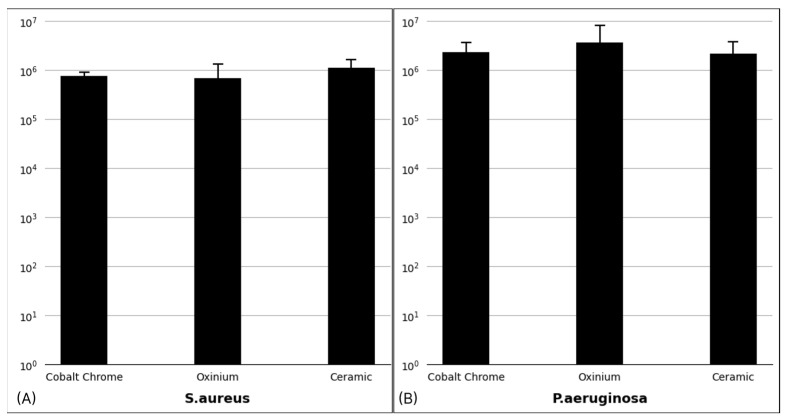
CFU counts of *S. aureus* (**A**) and *P. aeruginosa* (**B**) on different implant materials (cobalt–chrome, oxinium, and ceramic). Error bars represent standard deviations from triplicate experiments.

The mean CFU counts retrieved cobalt–chrome, oxinium, and ceramic for *S. aureus* were 7.6 × 10^5^ ± 9.7 × 10^4^, 6.9 × 10^5^ ± 3.6 × 10^5^, and 1.1 × 10^6^ ± 3.0 × 10^5^, respectively (Figure 3A). The differences were not statistically significant (*p =* 0.200), suggesting no material-dependent effect on *S. aureus* biofilm formation.

For *P. aeruginosa*, the mean CFU counts retrieved from cobalt–chrome, oxinium, and ceramic were 2.3 × 10^6^ ± 7.2 × 10^5^, 3.7 × 10^6^ ± 2.5 × 10^6^, and 2.2 × 10^6^ ± 8.9 × 10^5^, respectively (*p =* 0.160) (Figure 3B). Although oxinium showed a higher mean CFU count, the differences among the materials were within one log difference, which is considered clinically insignificant [22].

### 3.1. Biofilm Confirmation

**Figure 4:**
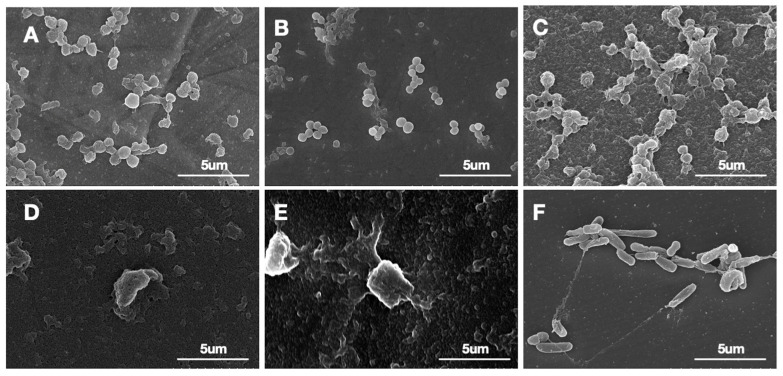
Representative images of the bearing surface of the femoral heads after bacterial culture. *S. aureus* biofilm was observed in all materials: (**A**) cobalt–chrome, (**B**) oxinium, and (**C**) ceramic, respectively. *P. aureginosa* was not observed on cobalt–chrome (**D**) and oxinium (**E**) femoral heads and while observed in the ceramic (**F**) femoral head, they were relatively less in number as compared to that of the *S. aureus*. Images were taken at a magnification of 10.0k×, with scale bars included for reference.

Scanning Electron Microscopy (SEM) assessing the biofilm on the bearing surface of the femoral head confirmed the presence of *S. aureus* biofilm on all tested materials. Regarding *P. aeruginosa*, no well-formed biofilm was observed on the bearing surface of cobalt–chrome and oxinium femoral head prostheses. While some bacterial remnants were detected, likely due to incomplete removal of planktonic bacteria during processing, there was no clear evidence of structured biofilm formation. In contrast, *P. aeruginosa* biofilm was observed on the ceramic surface, but only in small numbers (Figure 4).

### 3.2. Biofilm Distribution

**Figure 5:**
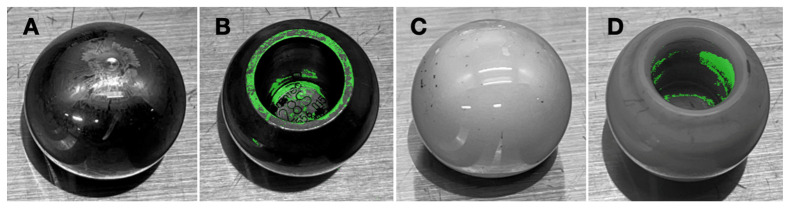
*S. aureus* biofilm distribution on the cobalt–chrome (**A**,**B**) and ceramic (**C**,**D**) femoral heads, respectively. The biofilm is not detected on the bearing surface of the femoral head (**A**,**C**) while a marked concentration is observed in the taper hole (**B**,**D**).

The crystal violet staining revealed an uneven distribution of biofilm across the femoral heads for *S. aureus*. A marked concentration of biofilm was observed around and within the femoral head taper hole, which indicates accumulation of biofilm in these regions. In contrast, the bearing surface of the femoral head showed nearly no staining (Figure 5). This pattern suggests a tendency for biofilm to accumulate in and around the taper area, potentially due to surface topography and to structural characteristic in these regions.

## 4. Discussion

The consistent results obtained from current study suggest that cobalt–chrome, oxinium, and ceramic materials do not differ significantly in their susceptibility to bacterial adherence and biofilm formation. Therefore, the material of the femoral head alone may not be a critical factor in preventing PJIs. This finding is generally in line with previous studies on orthopedic implant materials, which have similarly reported no significant material-dependent differences in biofilm formation. However, unlike these studies, the present study directly examined biofilm formation on actual contemporary femoral head prostheses [9,10,11]. This distinction suggests that while earlier findings remain valid, the current results provide a more direct assessment of biofilm behavior on femoral heads as used in clinical practice.

This is a clinically important finding, as it suggests that concern for biofilm formation should not be a primary consideration when selecting femoral head materials. Given the limited research in this area, clinicians typically base their decisions on factors such as mechanical properties, wear resistance, biocompatibility, and cost [23] (Table 1). Our study indicates that these factors remain primary, as the differences in biofilm susceptibility among these materials are not clinically relevant.

Previous research has extensively investigated the biofilm-forming capabilities of bacteria on various biomaterials across multiple fields, with dental and orthopedic implants being particularly prominent areas of study [26]. Within the orthopedic domain, numerous studies have focused on biofilm formation. Malhotra et al. investigated the biofilm adherence of *Staphylococcus aureus, Staphylococcus epidermidis*, *Escherichia coli*, *Klebsiella pneumoniae*, and *Pseudomonas aeruginosa* on five materials: cobalt–chromium, highly cross-linked polyethylene, stainless steel, trabecular metal, and titanium alloy. The highest adherence was observed on highly cross-linked polyethylene, followed by titanium, stainless steel, trabecular metal, and the lowest on cobalt–chromium alloy [9]. Additionally, Koseki et al. examined *Staphylococcus epidermis* on five materials—oxidized zirconium–niobium alloy, cobalt–chromium–molybdenum alloy (Co-Cr-Mo), titanium alloy (Ti-6Al-4V), commercially pure titanium (cp-Ti), and stainless steel—over incubation periods of 2, 4, and 6 h. They found no significant differences in the biofilm coverage rate (BCR) at 2 and 4 h, but after 6 h, the BCR for Co-Cr-Mo was significantly lower than that for Ti-6Al-4V, cp-Ti, and stainless steel, highlighting inconsistent results across time points [10]. However, these investigations employed simplified models, such as machined discs, which may not accurately replicate the complex clinical environment of actual implants [9,10]. The present study distinguishes itself by utilizing femoral head prostheses used in clinical surgeries. This approach provides a more realistic assessment of biofilm formation, addressing the unique challenges associated with orthopedic implants and enhancing the relevance of the findings to clinical practice.

The absence of significant differences in biofilm formation among the various prosthetic materials in our study can be attributed to the extremely smooth nature of the femoral head. Previous research has identified key factors influencing bacterial adhesion and biofilm development, including surface roughness, surface free energy (SFE), surface chemistry, and properties such as porosity, corrosion behavior, and the composition of surface materials [27]. Among these factors, surface roughness is often considered the most critical. Surface roughness, measured as Ra, is a key determinant in bacterial adhesion. A threshold Ra value of 200 nm is typically noted as significant for influencing bacterial adhesion [28]. The Ra values for cobalt–chrome, oxinium, and ceramic were reported to be 25 nm, 20–30 nm, and 20 nm, respectively [29]. These values are far below the 200 nm threshold, suggesting that the surface roughness of these materials do not contribute significantly to bacterial adhesion.

While surface roughness appeared to have minimal impact, the influence of surface free energy (SFE) and other surface characteristics or surface chemistry behavior remains complex. Previous studies have reported that polishing can alter multiple surface chemical properties, including SFE, and that increased SFE may contribute to enhanced bacterial adhesion. However, isolating the specific effects of SFE from other surface chemistry changes is challenging, as these factors are inherently interrelated and can be simultaneously affected by the polishing process [30]. Therefore, further research is needed to elucidate the distinct roles of SFE and surface chemistry in biofilm formation on these prosthetic materials. Nonetheless, the consistent biofilm densities observed across cobalt–chrome, oxinium, and ceramic femoral head prosthesis suggest that these factors do not have a major impact. Given that surface roughness has been established as a negligible factor and the combined variations in SFE and surface chemistry appear insufficient to alter biofilm adhesion meaningfully, it can be inferred that the inherent properties of these prosthetic materials do not differentially affect bacterial colonization. This inference aligns with our findings, reinforcing the notion that other variables may play more pivotal roles in biofilm formation and the subsequent risk of PJIs.

An additional important finding of the current study is regarding the location of where biofilm was formed on the femoral head prosthesis. Crystal violet staining revealed that biofilm formation was predominantly localized around and within the femoral head taper hole, rather than on the bearing surface. This finding has important clinical implications, particularly in the context of debridement, antibiotics, and implant retention (DAIR) procedures. Given the biofilm accumulation in the stem taper region, consideration should be given to replacing the femoral head during DAIR surgery when feasible. If replacement is not an option, careful removal and thorough cleaning of the existing femoral head, especially around the taper hole, may help reduce the risk of persistent infection.

### Limitations

We acknowledge there are a number of limitations to the current study. First of all, this is an in vitro study, and clinical interpretation should be made with caution. Numerous factors may influence formation of the biofilm in the inserted arthroplasty prosthesis, and therefore, the findings from the current study may be different in vivo.

A second limitation is regarding the choice of bacterial strains. Our study utilized two pathogens: *S. aureus* and *P. aeruginosa*, which are commonly responsible for PJIs [12]. However, the experiments were conducted using antibiotic-sensitive strains of both bacteria, which may not fully replicate the clinical PJI environment where antibiotic-resistant strains, such as Methicillin-Resistant *Staphylococcus aureus* (MRSA) and Coagulase-Negative Staphylococci (CoNS), are common [31]. In addition, *Staphylococcus epidermidis*, another common pathogen associated with biofilm-related implant infections, was not included in this study. Given its significant role in chronic PJIs due to its strong biofilm-forming capacity, future studies should incorporate *S. epidermidis* to provide a more comprehensive understanding of biofilm formation across different bacterial species. Furthermore, while PJIs are often caused by *S. aureus* and *P. aeruginosa*, acute hematogenous infections can also involve other bacterial species, such as *Escherichia coli* and *Streptococcus agalactiae*. Investigating these bacteria in future studies could offer valuable insights into the varying biofilm-forming capabilities of different pathogens and their implications in both early- and late-stage infections.

Another important limitation concerns the complexity of polymicrobial infections. While our study focused on monomicrobial biofilms of *S. aureus* and *P. aeruginosa*, PJIs often involve multiple bacterial species coexisting within the biofilm [31]. Previous studies have shown that interspecies interactions, including cell–protein interactions, primary and secondary metabolite exchanges, quorum sensing, and coaggregation, contribute to enhanced biofilm resilience and increased antibiotic resistance [32]. Such cooperative mechanisms among different microbial species can further complicate treatment strategies and reduce the efficacy of conventional antibiotics. Future studies incorporating polymicrobial biofilm models will be crucial to better understanding these interactions and their impact on antimicrobial resistance, ultimately leading to more effective therapeutic approaches for PJIs.

Additionally, femoral head prostheses, despite being wear-resistant, can develop surface scratches over time with actual use, for various reasons [33]. These scratches create a different environment from the initial experimental conditions, potentially increasing susceptibility to bacterial adhesion. In our study, the prostheses used were entirely smooth and unworn, differing from real clinical scenarios where surface wear and microabrasions occur over time. This may have influenced our findings, particularly regarding the lack of significant differences between the tested materials. Surface roughness is known to play a critical role in bacterial adhesion and biofilm formation, and thus, future studies should consider incorporating artificially worn or pre-scratched prostheses to better replicate in vivo conditions and assess their impact on bacterial colonization and biofilm development.

Moreover, many contemporary femoral head prostheses undergo surface modifications, such as specialized coatings or treatments, which may alter bacterial adhesion and biofilm formation. Since the prostheses used in this study did not include such modifications, the findings may not fully account for their potential effects. Future research should investigate how various surface treatments influence biofilm development, particularly in comparison to unmodified femoral heads, to better understand their role in infection prevention.

Moreover, our study focused solely on the femoral head and did not investigate non-mobile components such as the acetabular cup, polyethylene insert, or cemented and cementless stems. While the femoral head is a clinically relevant component frequently removed during DAIR procedures, we recognize that biofilm formation on other implant surfaces is also an important consideration. The different materials, surface characteristics, and fixation methods of non-mobile components and femoral stems may influence bacterial adhesion and biofilm development in ways not captured in our study. In particular, cemented and cementless stems may exhibit distinct biofilm formation patterns due to differences in surface roughness and the presence of bone cement, which were not explored in this study. Future research should expand on these findings by evaluating biofilm formation on a broader range of implant components, including both acetabular and femoral stem designs, to provide a more comprehensive understanding of PJIs.

Lastly, various factors present during arthroplasty surgery, such as exposure to blood or immune responses, were not accounted for in this study. In a clinical setting, the femoral head is also coupled with the femoral stem and liner within the patient’s body, which differs from our isolated experimental setup. Given that crystal violet staining revealed significant biofilm presence within the taper hole, this structural difference further suggests a potential gap between our experimental findings and actual clinical outcomes, as biofilm formation might be affected by the full assembly and in vivo conditions.

## 5. Conclusions

The results of the current study indicate that cobalt–chrome, oxinium, and ceramic materials show no clinically significant differences in their susceptibility to bacterial adherence and biofilm formation. As one of the few studies to assess the susceptibility of various materials used in contemporary femoral head prostheses, this study suggests that the material of the femoral head itself is not a major factor in preventing PJIs. Consequently, selecting a specific material type of the femoral head prosthesis may not be necessary for the purpose of preventing biofilm development and associated infections.

Furthermore, this study provides a scientific basis for the establishment of clinical guidelines regarding the selection of femoral head prostheses. Given that material choice does not contribute to infection prevention, future research should focus on the development of antimicrobial coating technologies to enhance the resistance of implants to bacterial colonization. In addition, clinicians should prioritize comprehensive infection control measures before and after surgery to minimize the risk of PJIs.

## Figures and Tables

**Table 1:** Key characteristics of different implant materials.

Material	Reasons to Use	Reasons to Avoid
cobalt–chrome [24]	Resistance to mechanical failure cost effective	Releases metal ion Higher wear particle production
oxinium [25]	Wear resistance Reduces release of metal ions	Higher cost Limited long-term data
ceramic [24]	Wear and scratch resistance Lower wear particle High biocompatibility	Brittleness May make noise Expensive than CoCr heads

## Data Availability

The raw data of the current study will be made available by the authors upon request.
